# An Empirical Study on Low-Carbon: Human Resources Performance Evaluation

**DOI:** 10.3390/ijerph15010062

**Published:** 2018-01-03

**Authors:** Quan Chen, Sang-Bing Tsai, Yuming Zhai, Jie Zhou, Jian Yu, Li-Chung Chang, Guodong Li, Yuxiang Zheng, Jiangtao Wang

**Affiliations:** 1 Zhongshan Institute, University of Electronic Science and Technology of China, Zhongshan 528400, China; zschenquan@gmail.com (Q.C.); m13686147477@163.com (L.-C.C.); 2 Economics and Management College, Civil Aviation University of China, Tianjin 300300, China; gdli@cauc.edu.cn; 3 School of Economics and Management, Shanghai Institute of Technology, Shanghai 201418, China; 4 College of Tourism and Service Management, Nankai University, Tianjin 300071, China; 5 Business School, Nankai University, Tianjin 300071, China; 6 Research Center for Environment and Sustainable Development of the China Civil Aviation, Civil Aviation University of China, Tianjin 300300, China; 7 School of Economics & Management, Shanghai Maritime University, Shanghai 201306, China

**Keywords:** AHP model, low-carbon, human resources, green logistics, green environment, green operation

## Abstract

Low-carbon logistics meets the requirements of a low-carbon economy and is the most effective operating model for logistic development to achieve sustainability by coping with severe energy consumption and global warming. Low-carbon logistics aims to reduce carbon intensity rather than simply reduce energy consumption and carbon emissions. Human resources are an important part of the great competition in the logistics market and significantly affect the operations of enterprises. Performance evaluations of human resources are particularly important for low-carbon logistics enterprises with scarce talents. Such evaluations in these enterprises are of great significance for their strategic development. This study constructed a human resource performance evaluation system to assess non-managerial employees’ low-carbon job capacity, job performance, and job attitude in the low-carbon logistics sector. The case study results revealed that the investigated company enjoyed initial success after having promoted low-carbon concepts and values to its non-managerial employees, and the success was demonstrated by excellent performance in its employees’ job attitude and knowledge. This study adopts the AHP method to reasonably determine an indicator system of performance evaluation and its weight to avoid certain human-caused bias. This study not only fills the gap in the related literature, but can also be applied to industrial practice.

## 1. Introduction

The low-carbon economy, which is based on low energy consumption, low pollution, and low emissions, is increasingly discussed because of the effects of global warming. Developed Western countries actively promote energy-efficient, low-emissions policies to develop low-carbon technologies. Additionally, these countries have adjusted their policies in areas such as industry, energy, technology, and trade to seize the initiative and reach commanding heights in the low-carbon revolution [1,2].

Low-carbon logistics meets the requirements of a low-carbon economy and is the most effective operating model for logistic developments to achieve sustainability by coping with severe energy consumption and global warming [3,4]. Low-carbon logistics aims to reduce carbon intensity rather than reduce energy consumption and carbon emissions [2]. Essentially, low-carbon logistics can both meet the requirement of moderate socioeconomic growth and mitigate the pressure of energy demand and supply (i.e., energy consumption efficiency in the logistics sector) through methods such as planning, policymaking, rationalization, standardization, digitalization, and low-carbon technologies. As a high-end service industry, the logistics sector must develop digitized green logistics services to achieve a low-carbon economy.

Human resources are a crucial resource for logistics companies to participate in market competition and a key factor in business operations, and human resource performance evaluation is imperative for low-carbon logistics firms for which talent is scarce [2,4,5,6]. Correctly evaluating the performance of low-carbon talent is significant for the strategic development of low-carbon logistics firms. This study constructed a human resource performance evaluation system to assess non-managerial employees’ low-carbon job capacity, job performance, and job attitude in the low-carbon logistics sector. The system provides guidance concerning the current status of corporate development and enables firms to develop optimal human resource management and make decisions accordingly.

## 2. Human Resource and Performance Assessment System for Low-Carbon Logistics Industry

Human resources are not only the main labor force but also a scarce resource in an enterprise. The dearth of low-carbon human resources is particularly highlighted in the developing trend of the low-carbon economy. In contrast to typical enterprises, human resources in the low-carbon logistics sector exhibit unique characteristics as follows:

(1) Low-carbon technologies. The logistics system of an enterprise can be divided into procurement, operating, sales, recycling, and information systems, encompassing processes such as transport, inventory, distribution, handling, and packaging [7,8]. The transport of goods relies on network technologies to determine optimal shipping routes, and inventory management requires operations research theories to optimize inventory. Because low-carbon technologies support various subsystems in a logistics system, human resources in logistics require a certain expertise in low-carbon technologies;
(2) Low-carbon rationale. A logistics system requires continuous improvement and must account for both environmental and resource issues to form a symbiotic system promoting economic development and healthy energy consumption. Low-carbon usage is the latest trend in the logistics sector, and low-carbon rationale is an essential component of job skills possessed by human resources in logistics. Green practices can achieve economic benefits while protecting the environment [8];
(3) Global strategies. The future development trend of low-carbon logistics will encompass the entire supply chain rather than merely the logistics industry. Low-carbon logistics involves a series of processes, such as raw materials procurement, goods manufacturing, shipping, and packaging, and low-carbon practices that must be integrated into the entire supply chain to achieve cost effectiveness in a two-pronged approach. Enterprises require a substantial amount of capital investment to respond to the low-carbon trend, and their human resources departments must possess a strategic vision to convert the investment to increased output while forming competitive advantages for the organizations;
(4) Innovative minds. Because enterprises must develop themselves against competition and low-carbon enterprises exhibit knowledge- and talent-intensive features, competition among enterprises has evolved into a recruiting war. Innovation in novel technologies or innovative use of existing technologies drives the sustainability of low-carbon logistics corporations.

Employee performance evaluation is a formal assessment system of job performance that measures employees’ job behavior and outcomes through systematic approaches and principles. Performance evaluation is the communication between managers and employees, and the outcomes can directly affect numerous employee benefits such as pay raises, bonuses, promotion, and demotion. Performance evaluation is key to organizational development. A structured performance evaluation system emphasizes the contributing indicators while discarding those irrelevant to organizational development [9].

Generally, employee performance can be assessed in dimensions such as organizational commitment, job capacity, job performance, job attitude, and employee growth. Commonly used performance evaluation methods are divided into results-oriented and behavior-oriented methods. For example, the results-oriented methods include rating scales, management by objectives, and key performance indicators, whereas behavior-orientated methods include the critical incident technique, behavior observation scales, behaviorally anchored rating scales, and 360° feedback. Additionally, performance evaluation methods exist that focus on employees’ traits (e.g., a graphic rating scale) [10].

This study specifically designed a human resource performance evaluation system for the low-carbon logistic industry. An analytic hierarchy process (AHP) was employed to measure non-managerial employees’ low-carbon job capacity and performance.

## 3. Research Methodology

### 3.1. Constructing an Analytic Hierarchical Process System

Company T is a large logistics firm located in Shenzhen, China. The firm has been committed to the development of low-carbon logistics in recent years and has achieved some success. Company T wants to understand its own utilization of low-carbon human resources and whether room for improving low-carbon human resource performance exists. This study used Company T as the subject of its case study and constructed a low-carbon human resource performance evaluation system to assess the company’s human resource performance.

**Figure 1:**
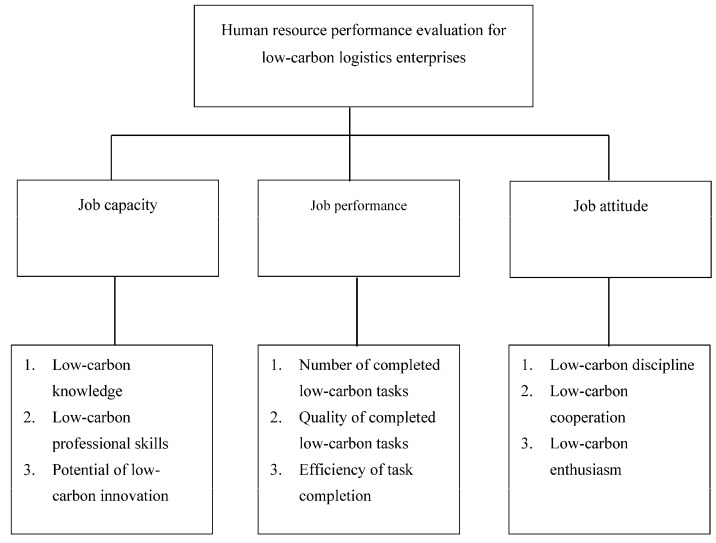
Human resource performance evaluation system for low-carbon logistics enterprises.

Figure 1 illustrates the hierarchical framework of the research objective, evaluating dimensions, and evaluating indicators according to the aforementioned discussion. The focus of this study was assessing the job capacity and outcomes of human resources in the low-carbon logistics industry. This study assessed employee performance using three dimensions, namely job capacity, job performance, and job attitude.

First, in accordance with AHP principles, the performance indicators in the low-carbon logistics industry were divided into the three aforementioned dimensions as primary indicators that were further divided into several secondary indicators. The secondary indicators of job capacity consisted of low-carbon knowledge, low-carbon professional skills, and potential of low-carbon innovation; those of job performance comprised the number of completed low-carbon tasks, the quality of completed low-carbon tasks, and the efficiency of task completion; and those of job attitude can be divided into low-carbon discipline, cooperation, and enthusiasm, as shown in Figure 1 and Table 1.

### 3.2. Questionnaire Design

The Level 1 for this questionnaire is the human resource performance evaluation of a low-carbon logistics enterprise; Level 2 contains three measurement indicators, which requires conducting pairwise comparisons three times (namely the pairwise comparison by using n(n − 1)/2 times); Level 3 also contains three evaluation indicators, and similarly each dimension must conduct pairwise comparisons three times. The focus of this study was assessing the job capacity and outcomes of human resources in the low-carbon logistics industry. This study assessed employee performance using three dimensions, namely job capacity, job performance, and job attitude.

First, in accordance with AHP principles, the performance indicators in the low-carbon logistics industry were divided into the three aforementioned dimensions as primary indicators that were further divided into several secondary indicators. The secondary indicators of job capacity consisted of low-carbon knowledge, low-carbon professional skills, and potential of low-carbon innovation; those of job performance comprised the number of completed low-carbon tasks, the quality of completed low-carbon tasks, and the efficiency of task completion; and those of job attitude can be divided into low-carbon discipline, cooperation, and enthusiasm, as shown in Table 1 (please refer to the aforesaid Figure 1 and Table 1).

This research used the aforesaid indicators to create the AHP questionnaire, where the measurement scale adopts a 9-point scale: “9” represents the greatest effect, “0” represents no effect, and the points between are scored according to the importance degree. The questionnaire survey was conducted during the period from 10 March 2017 to 20 April 2017. These authors visited 22 experts, including 12 professors, five senior managers of logistics enterprise, and five relevant government officials. A total of 22 valid questionnaires are retrieved, and there are no invalid questionnaires. After the explanation of the questionnaire contents, the questionnaires are delivered to the respondents to complete, and the valid questionnaire recovery rate is 100%.

### 3.3. Theoretical Foundation of AHP

AHP was developed by Saaty in 1971, and its main applications are uncertain situations and decision-making problems with multiple evaluation criteria. The hierarchical structure contributes to the understanding of objects and events that require decision-making. However, during the selection of appropriate options, each option must be assessed for certain benchmarks to determine the priority of available options and identify the appropriate solutions [11,12]. The AHP collects the opinions of scholars, experts, and participating decision makers through group discussion, and reduces a complex decision-making assessment system into a simple hierarchical system. A nominal scale is employed for pairwise comparison between elements in various classes. A pairwise comparison matrix is formulated after quantization, and the obtained eigenvectors serve as the priority vectors of the corresponding classes. Subsequently, maximized eigenvector comparison verifies the consistency of the matrix and provides decision makers with a decision-making reference [13,14,15].

The AHP initially lists all factors related to a decision-making problem before constructing an interrelated hierarchy. This method assesses elements in a class according to the effect of the elements above them in the hierarchy. That is, the relative contributions or importance (i.e., relative weight) of two factors in the same class are individually evaluated on the basis of the factors above them in the hierarchy. The pairwise comparison process decomposes a complex problem and enables evaluators to concentrate on the crucial relationship between two factors instead of encountering multiple factors at once [13]. The assumptions during AHP implementation are listed as follows [16,17,18]:

(1) A system or problem can be decomposed into numerous measurable classes or components that form the hierarchical structure of a directional network.
(2) The independence of elements in each hierarchical class is presumed, and some or all elements in the class above can be used as the benchmark for evaluation in the class underneath.
(3) During the evaluation, the absolute numerical scale can be converted into a ratio scale.
(4) After pairwise comparison, the reciprocal of the matrix is symmetric to the main diagonal and can be processed through a positive reciprocal matrix.
(5) The preference relations satisfy with transitivity. However, because perfect transitivity is difficult to achieve, intransitivity is allowed as long as the consistency is tested.
(6) The proportions of elements (i.e., priority) are obtained through weighting principles.
(7) Any element in the hierarchical structure is presumably relevant to the evaluated goal regardless of its priority weight.

### 3.4. AHP Applications

The AHP uses procedures and principles to summarize the evaluated scores and derive the priority of solutions and alternatives. AHP applications are implemented in three steps as follows [19,20,21,22]:

Step 1: Establish evaluating factors and a hierarchical structure

(1) Define the problem: Before a decision is made, individuals or groups should expand the decision-making system and describe the problem as much as possible. The scope and limitations of the problems should be defined and standardized before a decision is made.
(2) List the factors to be evaluated: All potential decision-making factors should be listed individually. For group decision-making, these factors can be listed through brainstorming, past experience, or research reports and serve as a reference for decision makers.
(3) Establish a hierarchical structure: The hierarchical framework does not have a fixed procedure for construction, but the highest and lowest classes are always the ultimate goal and alternatives of the evaluation, respectively. Elements with similar importance should be placed in the same class, and each class should not exceed seven elements. Additionally, all elements in a class should be independent from each other.

Step 2: Calculate weights of elements in each class

(1) Questionnaire design

This study designed a questionnaire according to the content of and relationships among various classes in the hierarchy. Pairwise comparison was conducted according to the benchmark set by the elements in a class above and supplemented by a scale ranging 1–9. The five basic scales consisting of equal importance, weak importance, essential importance, very strong importance, and absolute importance were assigned the numerical values 1, 3, 5, 7, and 9, respectively. Additionally, four intermediate values, comprising 2, 4, 6, and 8, lay between these five basic scales, as shown in Table 2.

(2) Construct a pairwise comparison matrix

The pairwise comparisons of elements from the same class were evaluated using a nominal scale and the benchmark of the evaluated elements from the class above. If n criteria exist, a total of n(n − 1)/2 pairwise comparisons are required. After all members of the decision-making group circle the importance of every element, the pairwise comparison matrix is formulated according to the results of the questionnaire. The comparison results of n criteria are placed on the upper triangle in the matrix, whereas the lower triangle comprises the reciprocals of the corresponding values on the upper triangle. The diagonal of the matrix contains only values of 1 [23,24,25,26].

(3) Verify the class consistency

After the eigenvalues and eigenvectors in the pairwise comparison matrix are calculated, the weights of elements in various classes can be obtained. Subsequently, the priority vectors can be identified through commonly used eigenvalue decomposition methods in numerical analysis. Saaty proposed four eigenvalue approximate solution methods in 1996 as follows [27,28,29]:

(a) Average of normalized columns.
(b) Normalization of the row average.
(c) Normalization of the geometric mean of the rows (NGM).
(d) In practice, the normalization of row vectors and their reciprocals uses the three aforementioned methods, with NGM being the most commonly used method. This study also employed NGM to obtain the weight of each evaluated criteria, and the formula can be expressed as follows:
  (1) $${\;W}_{i}=\frac{{\left(\prod_{j=1}^{n}a_{\mathrm{ij}}\right)}^{\frac{1}{n}}}{\sum_{i=1}^{n}{\left(\prod_{j=1}^{n}a_{\mathrm{ij}}\right)}^{\frac{1}{n}}}\;i,\;j=1,\;2,\;\ldots ,\;n$$

The calculation of the maximized eigenvalue (λmax) is explained as follows [30,31,32,33,34,35,36]: first, the pairwise comparison matrix A is multiplied by the eigenvector W to obtain a new eigenvector W′, wherein each eigenvalue is divided by the corresponding eigenvalue in W. Subsequently, the mean of the obtained values represents λmax.

Step 3: Calculate the weight of the entire class

After the weights of criteria in each class are calculated, those in the entire hierarchy are calculated. When there is only one decision maker, only comprehensive evaluation (i.e., priority) of the alternatives must be obtained. In a decision-making group, all alternatives proposed by various members must be calculated separately. Subsequently, the weighted square number is obtained through weighted mean methods (e.g., simple additive weighting approach) to determine the priority of alternatives [14].

## 4. Results and Discussion

### 4.1. Results

#### 4.1.1. Weight Calculation in Level 2

The three indicators in Level 2 consisted of job capacity (X_1_), job performance (X_2_), and job attitude (X_3_). The CR value obtained from the pairwise comparison matrix was 0.008 (CR < 0.1), indicating the consistency of the pairwise comparisons in Level 2. Table 3 lists the weights and ranks of each indicator.

Table 3 demonstrates that job performance exhibited the highest weight in Level 2 (0.472), followed by job capacity (0.331) and job attitude (0.197). Job performance accounted for approximately 50% of the overall weight, indicating its influence in the evaluation of human resource performance for low-carbon logistics enterprises.

#### 4.1.2. Weight Calculation in Level 3

The indicators in Level 3 were sub-factors extended from the indicators in Level 2. The secondary indicators of job capacity (X_1_) consisted of low-carbon knowledge (X_11_), low-carbon professional skills (X_12_), and potential of low-carbon innovation (X_13_). The weights of these three secondary indicators were 0.471, 0.265, and 0.264, respectively. The CR value obtained from the pairwise comparison matrix was 0.005 (CR < 0.1), indicating the consistency of pairwise comparisons.

The secondary indicators of job performance (X_2_) consisted of number of completed low-carbon tasks (X_21_), quality of completed low-carbon tasks (X_22_), and efficiency of task completion (X_23_). The weights of these three secondary indicators were 0.473, 0.277, and 0.250, respectively. The CR value obtained from the pairwise comparison matrix was 0.003 (CR < 0.1), indicating the consistency of pairwise comparisons.

The secondary indicators of job attitude (X_3_) consisted of low-carbon discipline (X_31_), low-carbon cooperation (X_32_), and low-carbon enthusiasm (X_33_). The weights of these three secondary indicators were 0.407, 0.387, and 0.206, respectively. The CR value obtained from the pairwise comparison matrix was 0.004 (CR < 0.1), indicating the consistency of pairwise comparisons. Table 4, Table 5 and Table 6 list the weights and ranks of the secondary indicators in Level 3.

#### 4.1.3. Consistency Test of the Entire Hierarchy

The consistency test of the entire hierarchy used CRH to determine the importance of each evaluated element in all dimensions and whether contradictions exist. CRH is obtained by dividing CIH by RIH. The calculated results using Equations are listed as follows:

CIH = 0.472 × 0.008 + 0.472 × 0.004 + 0.472 × 0.002 + 0.472 × 0.003 = 0.00802

RIH = 0.472 × 0.58 + 0.471 × 0.58 + 0.463 × 0.58 + 0.401 × 0.58 = 1.04806

CRH = 0.00802/1.04806 = 0.0076

The calculated CRH at 0.0076 was substantially lower than the standard value of 0.1, indicating the consistency of the entire hierarchy and high reliability of the relative weights of all indicators.

#### 4.1.4. Summary of All Weights

After all weights in respect to the corresponding level were assigned, the weights in Level 3 were multiplied by the corresponding weight in Level 2 to signify the importance in the entire evaluation model. Table 7 shows the weights relative to the level weights, overall weights, and ranks of each primary and secondary indicator in the evaluation of human resource performance for low-carbon logistics enterprises.

This study revealed that the top five key indicators of human resource performance evaluation for low-carbon logistics enterprises were quality of completed low-carbon tasks (X_22_), followed by low-carbon professional skills (X_12_), efficiency of task completion (X_23_), number of completed low-carbon tasks (X_21_), and low-carbon knowledge (X_11_).

### 4.2. Discussion

To appraise the low-carbon human resource performance of Company T, this study invited 22 experts (12 professors, five managers, five government officials) to use the AHP indicators constructed in this study. The nine indicators in three dimensions were scored using a 5-point scale. Subsequently, the mean scores for each indicator were converted to the weighted scores, as shown in Table 8.

The total score of Company T was 3.29, indicating inadequate low-carbon human resource performance. The low score was mainly attributed to the low score in the highly weighted job performance dimension (X_2_). All three secondary indicators of X_2_ had relatively low initial mean scores, indicating that Company T must urgently improve its number, quality, and efficiency of completed low-carbon tasks. Additionally, Company T scored inadequately for both low-carbon professional skills (X_12_) and potential of low-carbon innovation (X_13_) in the job capacity dimension (X_1_) but scored fairly well in low-carbon knowledge (X_11_) in the same dimension. In the job attitude dimension (X_3_), Company T scored remarkably in all three secondary indicators (X_31_–X_33_).

The case study revealed that Company T enjoyed initial success after having promoted low-carbon concepts and values to its non-managerial employees, and the success was demonstrated by the excellent performance in employees’ job attitude and knowledge. However, the early results were still inadequate because these employees performed insufficiently in low-carbon professional skills, low-carbon innovation potential, and job performance. Company T must implement and strengthen these inadequately performed indicators to improve its low-carbon human resource performance.

## 5. Conclusions

The academic contribution of this research is the establishment of a set of low-carbon human resource performance evaluation system, which can measure the enterprise employees’ indicators of job capacity, job performance, and job attitude, as related to low carbon. Thus, the enterprise can have a better understanding of its development status and improve their development plans to further make the best low-carbon human resource management and development decisions, which can guide the future development of the human resources of low-carbon enterprises. This research can provide some enlightening thoughts to research scholars, enterprises, and governmental agencies. Moreover, it can provide some research methods and research path references to subsequent relevant research regarding low-carbon human resources.

In addition to the theoretical contributions, the findings in this study can be practically applied to the logistics sector. However, we recommend that prospective researchers select additional industries and cases for further discussion in future research. Furthermore, other evaluation methods may be incorporated for analyses and comparisons in future research.

## Figures and Tables

**Table 1:** Performance evaluation system.

Primary Indicator	Weight	Secondary Indicator	Weight
X_1_ Job capacity	U_1_	X_11_ Low-carbon knowledge	U_11_
		X_12_ Low-carbon professional skills	U_12_
		X_13_ Potential of low-carbon innovation	U_13_
X_2_ Job performance	U_2_	X_21_ Number of completed low-carbon tasks	U_21_
		X_22_ Quality of completed low-carbon tasks	U_22_
		X_13_ Efficiency of task completion	U_23_
X_3_ Job attitude	U_3_	X_14_ Low-carbon discipline	U_31_
		X_32_ Low-carbon cooperation	U_32_
		X_33_ Low-carbon enthusiasm	U_33_

**Table 2:** Definition and explanation of AHP evaluation scale.

Scale	Definition	Explanation
1	Equal importance	The two compared elements have equal contributions to the problem
3	Weak importance	Experience and judgment moderately lean toward a certain element
5	Essential importance	Experience and judgment strongly lean toward a certain element
7	Very strong importance	The evidence of the preference of a certain element is very strong
9	Absolute importance	The evidence of the preference of a certain element is extremely strong
2, 4, 6, 8	Intermediate values	When a compromise value is needed

**Table 3:** Weights of indicators in Level 2.

Indicator	Weight	Rank
X_1_ Job capacity	0.331	2
X_2_ Job performance	0.472	1
X_3_ Job attitude	0.197	3
CI = 0.008; CR = 0.008		

**Table 4:** Weights of secondary indicators of job capacity (X_1_).

Indicator	Weight	Rank
X_11_ Low-carbon knowledge	0.265	2
X_12_ Low-carbon professional skills	0.471	1
X_13_ Potential of low-carbon innovation	0.264	3
CI = 0.004; CR = 0.005		

**Table 5:** Weights of secondary indicators of job performance (X_2_).

Indicator	Weight	Rank
X_21_ Number of completed low-carbon tasks	0.261	3
X_22_ Quality of completed low-carbon tasks	0.463	1
X_23_ Efficiency of task completion	0.276	2
CI = 0.002; CR = 0.003		

**Table 6:** Weights of secondary indicators of job attitude (X_3_).

Indicator	Weight	Rank
X_31_ Low-carbon discipline	0.206	3
X_32_ Low-carbon cooperation	0.407	1
X_33_ Low-carbon enthusiasm	0.387	2
CI = 0.003; CR = 0.004		

**Table 7:** Summary of weights of indicators in human resource performance evaluation for low-carbon logistics enterprises.

Level	AHP Indicators				
	Indicator		Level Weight (L)	Overall Weight (G)	Rank
1	Human resource performance evaluation for low-carbon logistics enterprises		1	1	-
2	X_1_ Job capacity		0.331	0.331	2
	X_2_ Job performance		0.472	0.472	1
	X_3_ Job attitude		0.197	0.197	3
3	X_1_ Job capacity	X_11_ Low-carbon knowledge	0.265	0.088	5
		X_12_ Low-carbon professional skills	0.471	0.156	2
		X_13_ Potential of low-carbon innovation	0.264	0.087	6
	X_2_ Job performance	X_21_ Number of completed low-carbon tasks	0.261	0.123	4
		X_22_ Quality of completed low-carbon tasks	0.463	0.219	1
		X_23_ Efficiency of task completion	0.276	0.130	3
	X_3_ Job attitude	X_31_ Low-carbon discipline	0.206	0.041	9
		X_32_ Low-carbon cooperation	0.407	0.080	7
		X_33_ Low-carbon enthusiasm	0.387	0.076	8

**Table 8:** Human resource performance evaluation of Company T.

Dimension	Indicator	Initial Mean Score	Rank of Mean Score	Overall Weight (G)	Overall Weight Rank	Score
X_1_ Job capacity	X_11_ Low-carbon knowledge	3.86	3	0.088	5	0.34
	X_12_ Low-carbon professional skills	3.11	7	0.156	2	0.49
	X_13_ Potential of low-carbon innovation	2.42	9	0.087	6	0.21
Subtotal				0.331		1.04
X_2_ Job performance	X_21_ Number of completed low-carbon tasks	3.58	5	0.123	4	0.44
	X_22_ Quality of completed low-carbon tasks	2.75	8	0.219	1	0.60
	X_23_ Efficiency of task completion	3.38	6	0.130	3	0.44
Subtotal				0.472		1.48
X_3_ Job attitude	X_31_ Low-carbon discipline	3.96	2	0.041	9	0.16
	X_32_ Low-carbon cooperation	3.82	4	0.080	7	0.31
	X_33_ Low-carbon enthusiasm	4.02	1	0.076	8	0.31
Subtotal				0.197		0.77
Total						3.29
